# Abstracts/Sommaires/Zusammenfassungen

**DOI:** 10.1155/S1463924681000345

**Published:** 1981


### Abstracts Sommaires Zusammenfassungen

### Page 119

**It is time to examine process stream analysis**
D. P. Manka

The advantages and advances in continuous analysis of process streams are discussed. Details are given of the sample process itself together with criteria for the selection of the analyser. Specific applications of process analysis in the petroleum and other industries are also discussed.

**Il est temps d'étudier l'analyse continue de processus**

Les progrès et les avantages de l'analyse continue de processus sont discutés. Des détails sont donnés pour le processus lui-même, ainsi que pour les critères déterminant le choix de l'analyseur. Des applications spécifiques de l'analyse continue dans l'industrie pétrolière et d'autres sont discutées.

**Es ist Zeit, die Prozesskontinuierliche-Analyse zu überprüfen**

Die Vor- und Nachteile der kontinuierlichen Analyse. im Prozessfluss werden diskutiert. Es werden Details angeführt für die Probenahme zusammen mit den Kriterien für die Auswahl des Analysators. Spezifische Anwendungen der Prozessanalytik in den Petro- und anderen Industrien werden ebenfalls diskutiert.

### Page 122

**Evaluation of a programmable analyser - the Vitatron PA800.**
J. M. Smith, *et al*

A report on the evaluation of the Vitatron PA800 analyser is presented. The instrument was assessed by its performance of six methodologies, using different principles of analysis. Additional studies were carried out to assess the precision and accuracy of the instrumental components. The analyser performed well during the assessment period and is recommended for use in the clinical laboratory.

**Evaluation d'un analyseur programmable — le Vitatron PA800**

Un rapport sur l'évaluation de l'analyseur Vitatron PA800 est présenté. L'instrument était jugé d'après sa performance en six méthodologies, en utilisant différents principes d'analyse. D'autres études ont été effectuées pour vérifier la précision et l'exactitude des différents composants de l'instrument. L'analyseur a montré de belles performances pendant la période de vérification et peut être recommandé pour l'utilisation dans le laboratoire clinique.

**Evaluation eines programmierbaren Analysators — der Vitatron PA800**

Es wird ein Bericht über eine Evaluation des Vitatron PA800 Analysators präsentiert. Es wurde die Leistung des Geräts mit sechs Methodologien beurteilt, die alle verschiedene Analysenprincipien beinhalten. Zusätzliche Studien wurden durchgeführt für die Beurteilung der Präzision und Genauigkeit der instrumentellen Baugruppen. Der Analysator zeigte während der Versuchszeit eine gute Leistung und kann für die Verwendung im klinischen Labor empfohlen werden.

### Page 128

**An evaluation of the Chemispek multichannel analyser**
R. W. Logan *et al*

An evaluation of a new British-manufactured multichannel analyser, the Chemispek, is described. The instrument is of the continuous flow variety and, in the configuration tested, analysed sodium, potassium, chloride, carbon dioxide, urea, creatinine, calcium and phosphate requiring a plasma volume of 260 µl. The instrument uses a microprocessor for data capture and operates at a speed of 120 samples per hour.

**Une évaluation de l'analyseur multicanal Chemispek**

L'évaluation du nouvel analyseur multicanal Chemispek de fabrication anglaise est decrite. L'instrument est du type à débit continu et permettait dans la configuration étudiée, d'analyser sodium, potassium, chlorure, dioxide de carbone, urée, créatinine, calcium et phosphate avec un volume de plasma de 260 µl. L'instrument utilise un microprocesseur pour collection des données et opère à une vitesse de 120 échantillons par heure.

**Eine Evaluation des Chemispek Vielkanal-Analysators**

Eine Evaluation des neuen, in England hergestellten Vielkanal-Analysators Chemispek wird beschrieben. Das Instrument arbeitet mit kontinuierlichem Fluss und analysiert in der geprüften Konfiguration Natrium, Kalium, Chlor, Kohlenstoffdioxyd, Harnstoff, Kreatinin, Kalzium und Phosphat und benötigt ein Plasmavolumen von 260 µl. Das Instrument macht Gebrauch eines Mikroprozessors für die Datenerfassung und arbeitet mit einer Geschwindigkeit von 120 Proben pro Stunde.

### Page 134

**Increasing the analysis rate of automatic analysers by subtractively correcting for specimen interaction**
J. H. T. Bates *et al*

Automatic analysers are used routinely in hospital biochemistry laboratories in the analysis of biological specimens. In order to analyse specimens at a rate acceptable for routine use the analyser response peaks are made to partially overlap so that some means of correcting for the resulting specimen interaction becomes necessary. A subtractive correction method is presented, which is applicable when the shape of each peak is the same to within a vertical scaling factor. The subtractive method is compared to the commonly used carryover correction method and is shown to allow a considerable increase in the rate of analysis of specimens for comparable accuracy.

**Augmentation de la vitesse d'analyse d'analyseurs automatiques par correction substractive d'interactions de spécimens**

Des analyseurs automatiques sont utilisés de façon routinière dans les laboratoires biochimiques d'hôpital pour l'analyse de spécimens biologiques. Afin d'analyser des échantillons à une vitesse acceptable pour la routine, l'on fait que les pics se recouvrent partiellement, ce qui oblige de corriger pour l'interaction qui en résulte. Une méthode de correction subtractive est présentée, qui s'applique quand la forme de tous les pics est identique dans le cadre d'une échelle verticale. La méthode subtractive est comparée à la méthode habituelle de correction pour contamination et il est montré qu'elle permet une vitesse d'analyse nettement plus importante avec une exactitude comparable.

**Erhöhung des Probendurchsatzes von automatischen Analysengeräten durch Korrektur der Wechselwirkung aufeinanderfolgender Proben**

Automatische Analysengeräte werden routinemässig in Spital-Biochemielaboratorien für die Analyse biologischer Proben benützt. Um Proben mit einem Druchsatz zu analysieren, der für die Routine akzeptierbar ist, werden die Analysenpeaks teilweise zum Ueberlappen gebracht, so dass eine Korrektur für daraus resultierende Probenwechselwirkung notwendig wird. Es wird eine subtraktive Korrekturmethode vorgestellt, welche anwendbar ist, falls die Peakform abgesehen von einem Multiplikationsfaktor immer gleichartig ist. Die subtraktive Methode wird verglichen mit der normalerweise benützten Verschleppungskorrekturmethode, und es wird gezeigt das bei vergleichbarer Genauigkeit eine erhebliche Erhöhung der Druchsatzrate von Analysenproben möglich ist.

### Page 138

**A microcomputer automated circular dichroism spectropolarimeter**
V.C. Zadnik, *et al*

A Cary 61 CD spectropolarimeter was automated with an Altair 8800 microcomputer. In addition to collection of CD data, the microcomputer was designed to control the scan rate and direction, the range and period switches, the chart recorder of the instrument, and the zero suppression. Circuits were also incorporated to measure the setting of the monochromator. Software was written to measure and record CD signals, and to also use these measurements in real-time to control the progress of the experiment.

**Un spectropolarimetre pour dichroisme circulaire (CD) automatisé par microordinateur**

Un spectropolarimètre CD Cary 61 a été automatisé avec un microordinateur Altair 8800. En plus de la collection des données CD, le microordinateur contrôle la vitesse et la direction d'enregistrement, les commutateurs de domaine et de période, l'enregistreur de l'instrument et la suppression du zéro. Des circuits incorporés permettent de mesurer le positionnement du monochromateur. Le software a été écrit pour mesurer et enregistrer les signaux CD, mais également pour utiliser ces mesures en temps réel pour contrôler le progrès de l'expérience.

**Automatisiertes Spektropolarimeter für Rotations-Dichroismus**

Ein Cary 61 CD Spektropolarimeter wurde mit einem Altair 8800 Mikrocomputer automatisiert. Neben der Erfassung der CD Daten wurde dem Mikrocomputer die Steurerung der Abtasträte und Abtastgeschwindigkeit, des Bereichs und der Periodenschalter, des zum Instrument gehörigen Schreibers und der Nullpunktunterdrückung übertragen. Auch wurden Schaltungen eingebaut, die die Position des Monochromators messen. Die Software misst und schreibt die CD Signale, vermag aber auch diese Messungen für die Echtzeitsteuerung des Experiments zu benützen.

### Page 148

**Interfacing analytical instruments to electronic data processing systems - a summary of problems**
H. Pangritz

Only aspects of data processing in clinical chemistry and haematology are considered; within this brief, problems of data acquisition, calculation of results and evaluation of series of data, such as in quality control, are all covered. The collection and presentation of data blocks to a central laboratory computer and the positioning of the interface with relation to the computer and the analyser are discussed in detail and this is followed with a consideration of the content of the data blocks and the problems associated with its collection. The author makes a case for standardising the configuration of data processing interfaces.

**Interfacer des instruments analytqiues à des systèmes de traitements de données - un résumé des problèmes**

Uniquement les problèmes en chimie clinique et en hématologie sont étudiés; ce résumé traite de tous les problèmes d'acquisition de données, de calculs de résultats et d'élaboration de séries de données, tels qu'on en rencontre en contrôle de qualité. La collection et présentation des blocs de données à un ordinateur central de laboratoire et la position de l'interface entre l'ordinateur et l'analyseur sont discutées en détail. Ceci est suivi d'une étude du contenu de ces blocs de données, ainsi que des problèmes associés avec leur collection. L'auteur s'emploie pour standardiser la configuration d'interfaces de traitement de données.

**Anschluss analytischer Instrumente an die elektronische Datenverarbeitung - Zusammenfassung der Probleme**

Es sind nur die Aspekte der klinischen Chemie und der Hämatologie berücksichtigt; in diesem Bereich werden die Probleme der Datenerfassung, Resultatberechnung und Evaluation von Datenmessungen, wie sie z.B. in der Qualitätskontrolle anfallen, behandelt. Die Zusammenstellung und Weitergabe von Datenblöcken an ein zentrales Labordatensystem und die Positionierung des Interface in Bezug auf Rechner und Instrument werden im Detail diskutiert und sind von einer Betrachtung zum Inhalt der Datenblöcke und der damit zusammenhängenden Probleme der Erfassung gefolgt. Der Autor argumentiert für die Standardisierung der Konfiguration des Datenverarbeitungs-Interface.

### Page 151

**An automatic densimeter system for the measurement of the alcoholic strength of potable spirits**
R.G. Lidzey et al

An automated sample feed for loading a densimeter cell with alcohol solutions is described. Density values which are printed out at a terminal are used for the measurement of alcohol strength. The solutions are pre-cooled and any bubbles of air are trapped before entering the cell. Results are compared with standard density bottle measurements.

**Un système de determination automatique de densité**

Un système automatique pour charger des échantillons alcooliques dans un densitomètre est décrit. Les valeurs de densité imprimées sur un terminal sont ultilisées pour mesurer la concentration alcoolique. Les solutions sont refroidies d'avance et d'éventulles bulles d'air sont éliminées avant d'entrer dans la cellule. Les résultats sont comparés avec des mesures standards de densité.

**Ein automatisches Dichtebestimmungs-System**

Es wird ein automatischer Probenwechsler für die Beschickung einer Dichtemesszelle mit Alkohol-Lösungen beschrieben. Die Dichtemesswerte, welche an der Datenstation ausgedruckt werden, werden für die Bestimmung des Alkohlgehalts benützt. Die Lösungen werden vorgekühlt, und Luftblasen werden vor dem Zufluss in die Zelle entfernt. Die Resultate werden mit Standard-Dichtemessungen verglichen.

### Page 155

**Enzymic determination of glucose with SMAC: adaptation of the dichlorophenol/aminophenazone chromogen**
F. G. Gella *et al*

An automated method involving the use of 4-aminophenazone as a colour coupler with sulphonated 2,4-dichlorophenol has been adapted to SMAC for the analysis of glucose. Hydrogen peroxide, produced when glucose is oxidised by glucose oxidase, forms an intensely coloured dye in the presence of peroxidase and the chromogenic oxygen acceptor. The method only needs one stable reagent that can be prepared and stored in powdered or lyophilized form. The glucose channel of the instrument needs only two minor modifications: substitution of the interference filter and disconnection of two reagent lines. The proposed method is reproducible and the results correlate well with those obtained by the manual hexokinase procedure.

**Détermination enzymatique de la glucose avec SMAC: adaptation du chromogène dichlorophénol/aminophénazone**

Une méthode automatique utilisant le 4-aminophénazone comme coupleur de couleur avec le 2,4-dichlorophénol sulphoné a été adaptée au SMAC pour l'analyse de la glucose. L'eau oxygénée, produite lors de l'oxidation de la glucose par l'oxidase de glucose, forme un colorant intense dans la presence de peroxidase et de l'accepteur chromogène d'oxygène. La méthode n'a besoin que d'un réactif stable qui peut être préparé et gardé sous forme pulvérisée ou lyophilisée. Le canal de glucose de l'instrument ne demande que deux modifications mineures: substitution du filtre d'interférence et disconnection de deux lignes de réactifs. La méthode proposée est reproductible et les résultats donnent une bonne corrélation avec ceux obtenus par la méthode manuelle avec hexokinase.

**Enzymatische Bestimmung von Glukose mit dem Technicon SMAC System: anpassung des Dichlorphenol/Aminophenazon Chromogens**

Eine automatische Methode, die 4-Aminophenazon als Kuppelfarbstoff mit sulfoniertem 2,4-Dichlorphenol benützt, wurde zur Bestimmung von Glukose an den SMAC adaptiert. Wasserstoffperoxyd, dass bei der Oxydation von Glukose zu Glukoseoxydase entsteht, bewirkt in der Gegenwart von Peroxidase und dem chromogenen Sauerstoffakzpetor eine intensive Färbung. Die Methode benötigt lediglich ein stabiles Reagens das in pulverisierter oder lyophilisierter Form hergestellt und gelagert werden kann. Der Glukosekanal des Instruments benötigt nur zwei kleinere Modifikationen: Substitution des Interferenzfilters und Entfernung zweier Reagensleitungen. Die vorgeschlagene Methode ist reproduzierbar, und die Resultate korrelieren gut mit denjenigen, die mit der manuellen Hexokinase-Methode erhalten werden.

